# College Notes

**Published:** 1896-10

**Authors:** 


### College Notes.

The dental department of the University of Buffalo opened its fifth regular session on Tuesday in the new building erected during the Summer vacation. The new building, which is directly back of the old quarters occupied by the dental department in the university building and separated from it by a court, is not entirely completed yet. The freshman quarters are habitable, however, and the furniture necessary for their work has been moved over and the regular work has begun.

The freshman class numbers between forty and fifty students, and will probably reach between seventy-five and eighty before the date of entry is closed. The junior and senior classes begin their work on October 23d.

The annual announcement of the Buffalo College of Pharmacy has been issued. The only change of importance is the appointment of J. George Meidenbauer, a graduate of the college, as instructor in pharmacognosy. Dr. Wende will resume his class in botany, which has been under Dr. Benedict for the past few years. The establishment of what is really a three years' course in the college, which leads to the degree of Phar. M., appears to have been a success. Four men completed one year of it last year, and several of the graduates will return this fall to complete the larger course.

The formal opening of the Medical Department of the Buffalo University began September 14, 1896, in the University building on High street. Dr. Matthew D. Mann, the Dean of the faculty, opened proceedings with a graceful speech of welcome, and the address was delivered by Dr. Woods Hutchinson, formerly of the Iowa State University, who assumes here the chair of comparative pathology.

---

THE fourteenth annual session of the Medical Department of Niagara University will begin Thursday, October 1, 1896, at 9 A. M.

